# Past, present, and future nuisance flooding on the Charleston peninsula

**DOI:** 10.1371/journal.pone.0238770

**Published:** 2020-09-18

**Authors:** James T. Morris, Katherine A. Renken

**Affiliations:** Belle Baruch Institute for Coastal and Marine Science, University of South Carolina, Columbia, South Carolina, United States of America; Technische Universiteit Delft, NETHERLANDS

## Abstract

A forecast of nuisance flooding of Charleston peninsula is presented, based on an analysis of tide records from Charleston Harbor, SC. The forecast was based on past trends in local sea level and tidal harmonics, including the 18.6-yr lunar nodal and annual cycles. The data document an exponential rise in mean sea level. Extrapolating to year 2060 shows that the sea-level trend already is equivalent to the RCP4.5 scenario and on track to exceed NOAA’s intermediate low sea-level rise scenario of 0.5 m this century. If the trend continues, MSL will have risen by 0.22 m in 50 yr at an annual rate of 0.5 cm/yr in 2069. Simulations to 2064–2068, based on an empirical relationship between the annual number of flood events, defined as a water level exceeding 1.17 m NAVD (North American Vertical Datum of 1988), and the annual sum of monthly mean high water (r^2^ = 0.84), predict annual flood events will rise to the 60 to 75 range. Application of the hourly tidal harmonics to the long-term sea-level trend provided estimates of total land area flooded and duration of flooding. Flood duration is expected to rise to 6.5% by 2046–2050 and 8.2% of time by 2064–2068. The area exposed to flooding will be 4.23 km^2^ in 2046–2050 and 4.46 km^2^ in 2064–2068, corresponding to about 20–21% of peninsular area on what was formerly marshland and creeks, filled in earlier centuries. Finally, the estimated cost of defending the city and a proposal for a climate tax are discussed.

## Introduction

Coastal cities are experiencing an increase in occurrences of nuisance flooding, and the cost of remediation is going to be high. Nuisance flooding has become more common because of the combination of sea-level rise, land subsidence, and development. The focus of this study, the City of Charleston, SC, is among the top 10 cities in total cost of adding sea walls, estimated to be over than $1 billion [1]. The problem is exacerbated by the continued population growth and development of the coastal zone. The population living at or near sea level continues to grow dramatically, and this growth is accompanied by an associated increase in infrastructure. Over 28% of South Carolina’s 5.02 million residents live in its eight coastal counties [2]. From 1970 to 2010, the coastal county population of South Carolina increased by 127%, third highest among the 31 coastal and Great Lakes states nationwide, and is expected to grow another 23% by 2020 [3].

Results of process-based models that incorporate ice-sheet modelling and ocean thermal expansion give ranges of likely sea-level rise depending on expected increases in global temperatures resulting from different greenhouse gas emission scenarios, referred to as Representative Concentration Pathways (RCPs). RCP2.6 assumes a peak in CO_2_ concentration between years 2010 and 2020 followed by a decline, while RCP8.5 assumes unfettered emissions to about 900 ppm CO_2_ by 2100 [4]. For the period 2081–2100, compared to 1986–2005, global mean sea-level rise is likely to be 0.26 to 0.55 m for RCP2.6, 0.32 to 0.63 m for RCP4.5, 0.33 to 0.63 m for RCP6.0, and 0.45 to 0.82 m for RCP8.5. For RCP8.5, the expected rise by 2100 will be 0.52 to 0.98 m with a rate during 2081–2100 of 8 to 16 mm/y. The sea-level rise scenarios adopted by NOAA are based on projections of 0.5 m (intermediate low) and 1.0 m (intermediate) by 2100 [5] based loosely on RCP4.5 and RCP8.5. Based on these sea-level rise (SLR) scenarios, Sweet et al. [6] estimated that by 2050, high tide flooding will occur on average about 25 and 85 days/yr, respectively, (35 and 65% from tides) along the Southeast Atlantic in general, and 45 and 115 days for Charleston in particular.

Sweet and Park [7] provided strong evidence of a nonlinear increase in coastal flooding and sea level over the last half-century. According to their data analysis, parts of Charleston flooded in 1950 about 2 days annually for a total of 4 hr. The annual number of flood days remained in the single digits for the next 38 years until 1988. By 2014 annual flood days had increased to 25 for a total of 42 hr. Extrapolating their model to 2051, the annual flood days was forecasted to rise to 60 days for a total of 103 hr. It is important to note that a flood is defined as having occurred with high water level exceeds a tidal datum, not by direct observation, and then only portions of Charleston flood because of its topography. Still, the evidence is clear that the incidence and duration of flooding are increasing in a nonlinear manner [7].

By reason of tourism, trade, and manufacturing, the City of Charleston is an economic engine for the State. Its port is one of the busiest and fastest growing container ports on the East and Gulf coasts, and in 2018 it ranked eighth in the nation for dollar value of international exports, with cargo valued at approximately $26 billion [8]. Tourists are drawn to its history and cultural resources. The City contains hundreds of historic buildings and a historic district of 1,785 acres of National Register sites [9]. The City drew 6.9 million visitors in 2017 with a $7.37 billion impact [10]. Its cultural resources are a national treasure and are irreplaceable. At the time of a 2008 report on preservation there were nearly 5,000 structures built between 1712 and 1945 in the historic district [11], yet the preservation plan did not mention rising sea level or flooding.

Regional and global scale projections of sea level and flooding are based on combinations of process and empirical models, all based on uncertain projections of greenhouse emissions, and these do not necessarily align with local conditions. Nuisance flooding is locally the product of eustatic sea-level rise, tectonic movements, wind, currents, and subsidence [12–17]. These can have additive or opposing effects and may explain why acceleration of rising sea level has not been detected in some regions. Detection also depends on the length of the record, noise, and the ability to filter decadal-scale cycles [18, 19].

To aid in coastal planning, site-specific projections of flooding are needed. In this paper we present the results of a flood model developed for the Charleston peninsula. It is a conservative, bottom up approach to forecasting based on past trends in local sea level and tidal harmonics. We constructed statistical models to predict 1) future mean monthly sea level, 2) the number of annual flood events, and 3) flood duration and spatial extent on the Charleston peninsula. Our forecasts are based on extrapolations of the current sea level trend in Charleston harbor. The forecasts extend for 50 years only and account for acceleration in sea-level rise by means of an exponential model. The model uses a Monte-Carlo technique with probabilities drawn from real observations. An alternative model based on the intermediate sea-level rise scenario is also presented.

## Models and methods

Three groups of models are presented below that we will later refer to collectively as the M-R model. First is an empirical model of sea-level rise based on the fit of an exponential model to the tidal datums, MLW, MSL, MHW and maximum water level. Also included is an alternative quadratic model used to extrapolate 50 yr into the future based on an assumption that sea level will rise 1 m in the next century. Next, we present a model of the annual number of flood events based on the calculation of monthly MHW, and it is used to forecast future flooding using the exponential extrapolation of MHW and a Monte Carlo procedure. Finally, a model of hourly tidal harmonics fitted to Charleston tide data is added to the exponential and quadratic monthly MSL projections to derive the total hours of flooding expected in future years, also using a Monte Carlo procedure. The water levels from the extrapolated hourly tidal harmonics were binned, and their exceedance frequencies were used to generate spatially distributed heat maps of future flood duration on the Charleston peninsula.

### The exponential sea-level model

Detection of sea-level acceleration requires removal of interannual to multidecadal variability in sea-level records [20], which was accomplished by filtering seasonal and decadal cycles using harmonic regression analysis. Both exponential and quadratic models of the long-term trend were fitted, and only the exponential model was statistically significant:

$$MSL_{\left(t\right)}=\left\lfloor \left(1+MSL_{(}{}_{0)})e^{r}{}^{t}+a_{1}sin(2\pi \text{t}/12+p_{1}\right)\right\rfloor -1$$ (1)

where MSL_(t)_ is the monthly mean water level recorded at the NOAA Charleston Harbor station 8665530 (m NAVD). As written, this model accounts for a seasonal change in MSL as a1sin(2πt12+p1) due to the solar annual cycle, as discussed below and as recommended [16]. MSL_(0)_ is a least-squares estimate of MSL at time zero (January 1900), *r* is a proportionality constant (the relative monthly rate of increase), and *t* is the cumulative number of months. The amplitude of the solar annual cycle (*a*_*1*_) was entered into the model as a known constant, discussed next, while MSL(0), *r*, and the phase shift *p*_*1*_ were derived by nonlinear least-squares analysis (SAS 9.4 PROC MODEL).

### The solar annual cycle

The solar annual cycle [21], a_1_sin(2πt/12+p_1_) in Eq 1, is a seasonal change in MSL due to the thermal expansion and contraction of the ocean due to changes in solar insolation, atmospheric pressure, prevailing wind, currents, and runoff [22–24]. This cycle is not astronomically forced and, consequently the timing of the cycle’s apex and its amplitude varies. The amplitude of solar annual cycle a_1_ was entered into the exponential model as a constant by computing the average of the differences between the annual maxima and minima of monthly MSL for years of uninterrupted data from 1991 through 2017. The mean and standard error of a_1_ were 0.16 ± 0.03 m.

### Mean high and low water levels

Monthly mean high (MHW_(t)_) and mean low water (MLW_(t)_) levels were modeled as an offset (a_H_ and a_L,_ respectively) to MSL, with the addition of the 18.6-yr lunar nodal cycle (Eqs 2 and 3). Each of these can be modelled as a function of time, but including MHW and MLW as a function of MSL in the same PROC MODEL procedure added additional constraints to the exponential fit of MSL to time.

### The lunar nodal cycle

This 18.6-year cycle derives from the inclination of the moon’s orbit around the earth to that of the earth around the sun. The declination of the lunar orbital plane changes relative to the Earth’s equator from 18° 18’ to 28° 36’ and back every 18.61 years [25, 26]. With the addition of this cycle the monthly MHW and MLW were computed:

$$MHW_{\left(t\right)}=MSL_{\left(t\right)}-a_{H\left(t\right)}+a_{2}sin[2\pi t/(12C_{L})+p_{2}]$$ (2)

$$MLW_{\left(t\right)}=MSL_{\left(t\right)}-a_{L\left(t\right)}+a_{3}sin[2\pi t/(12C_{L})+p_{2}]$$ (3)

where *a*_*H*(*t*)_ is the average amplitude or MHW-MSL, *a*_*L*(*t*)_ is the average MSL-MWL, and *a*_*2*_ and *a*_*3*_ are the higher and lower amplitudes of the lunar nodal cycle, respectively; i.e. they generate the cycle in MHW and MLW. *C*_*L*_ is the cycle length in years (theoretically 18.6 yr), and p_2_ is the phase shift. Parameters *a*_2_, *a*_3_, *p*_2_, *a*_*H*(*t*)_, *a*_*L*(*t*)_, and *C*_*L*_ were estimated by non-linear parameter estimation (S1 Table in S1 File).

### Monthly maximum water level

The maximum water level (*W*_max(*t*)_) by month and year was extracted from a time series of hourly data extending between October 1921 and the end of 2017, and modelled as a deviation w_x_ from the monthly mean high water level, MHW_(t)_, with a best fit value of 0.62 m (S2 Table in S1 File).

$$W_{\max\left(t\right)}=w_{x}+\;MHW_{\left(t\right)}$$ (4)

Models 1–4 were fitted to the time series with a SAS 9.4 PROC MODEL in a single procedure.

### Monte-Carlo simulations of monthly means

Monthly MSL, MHW, MLW, and W_max(t)_ forecasts were made using Monte-Carlo simulations with normal probability distributions derived from the standard deviations of the residuals of the observed and predicted water levels computed for the period 1900 to 2017 excluding missing data (S2 Table in S1 File). The Monte-Carlo procedure generates a random set of additive error values, one for each observation and each equation, and computes one set of perturbations for each time in the series. These perturbations were added to the predicted mean water levels in Eqs 1–4 to develop the simulated water levels, both past and 50-yr into the future.

### Annual days of nuisance flooding

From recorded hourly water levels during 1921–2017 we computed the number of days when the water levels exceeded the threshold for nuisance flooding (1.17 m NAVD 88). These were the ‘observed’ flood events. The MHW tidal datum from the 1983–2001 epoch is 0.69 m (NAVD). Of several models tested (S4 Table in S1 File), the best predictor of the number of annual flood events (*NF*) for the data available was the annual sum of mean monthly MHW:

$$NF=c_{1}\sum_{1}^{12}MHW_{t}\;\text{for}\;\sum_{1}^{12}MHW_{t}\;\geq 7$$ (5)

(See Fig 4 inset)

*NF* is the number of flood events per year, $\sum_{1}^{12}MHW_{t}$ is the annual sum of monthly MHW, and *c*_1_ is an empirical constant. To make the forecasts to 2068, the Monte-Carlo simulation with the exponential model (Eq 2) was used to compute the perturbed monthly MHW_(t)_, and the results summed by year to give annual totals, which were inputted to Eq 5.

### Hourly water level and exceedance duration

Exceedance durations (the proportion of total time water level exceeds a threshold) were computed from forecasts of mean monthly sea level (Eq 1) with hourly tidal harmonics superimposed. Hourly tide levels were from harmonic regressions fitted to hourly data spanning 2008 through 2017 (S5 Table in S1 File). We fitted a model accounting for five important tidal constituents—the principal lunar (M2) and solar constituents (S2), and the diurnal (K1), lunar diurnal (O1), and solar annual constituents (Sa). These were fitted stepwise to hourly water levels for each of 10 annual time series from 2008 to 2017, and the fitted constants for amplitudes and phase shifts averaged (S3 Table in S1 File).

Next, to sync the phase shifts, the annual hourly time series from 2012 through 2017 were concatenated and the model refitted with known amplitudes (from the means of the stepwise procedure) and with the addition of the exponential function (MSL_(0)_ e^(*r* t)^) with known constants MSL_(0)_ and *r* to account for the long-term trend:

$$MSL_{\left(h\right)}=0.6527{\text{e}}^{\left(3.21\;10^{-4}\;t\right)}+M2_{\left(h\right)}+S2_{\left(h\right)}+K1_{\left(h\right)}+O1_{\left(h\right)}+Sa_{\left(h\right)}+W_{corr}$$ (6)

where *t* and *h* are the cumulative months and hours since year 1900, respectively. Parameter *W*_*corr*_ was the intercept (0.027 m ± 0.001 SE) for the January 2012 water level (theoretically zero).

To forecast hours of flooding we used Eq 6 to simulate the tidal cycle by Monte-Carlo simulation with a probability distribution based on the standard deviation of the hourly residuals (ε_(h)_ mean 0, 1 SD = 0.297 m) and that of the monthly exponential model (ε_(t)_ mean 0, 1 SD = 0.095 m). Then hourly water levels from the Monte Carlo forecasts of years 2012–2018, 2046–2050, and 2064–2068 were binned into 0.05 m groups, and cumulative frequency distributions were computed (SAS 9.4 Proc FREQ) for each group (S6 Table in S1 File). Each bin contained the percentage of total hours that water levels reached that elevation or higher during each sequence of years. From the frequency distributions we calculated the proportion of time that water level exceeded the threshold flood level of 1.17 m NAVD for nuisance flooding in Charleston, defined by Sweet et al. [7] as a water level exceeding 0.38 m above MHHW (1.18 m NAVD), relative to the 1983–2001 epoch. From these frequencies we generated heat maps that were mapped onto the Charleston DEM.

### The forward looking quadratic MSL model

Results of the exponential model forecast were compared to NOAA’s Intermediate scenario of a projected acceleration of SLR to 1 m by 2100. Although a quadratic model (Eq 7) did not give a satisfactory fit to past water level data from Charleston (S3 Table in S1 File), going forward an acceleration was modelled using a formula adopted by the NRC [27] and USACE [28]:

$$MSL_{\left(t\right)}=MSL_{\left(0\right)}+c_{2}t+\;c_{3}t^{2}$$ (7)

MSL_(0)_ was taken to be the mean water level in 2012 (= 0.01 m NAVD), *t* is the elapsed time (months) since 2012, and $c_{2}=\left(MSL_{\left(1\right)}-MSL_{\left(0\right)}\right)-\;c_{3}=2.9\;10^{-4}$. Substituting for *c*_*2*_ and solving for *c*_*3*_ gave:

$c_{3}=\left[\left(MSL_{\left(1200\right)}-MSL_{\left(0\right)}\right)/t-\;\left(MSL_{\left(1\right)}-MSL_{\left(0\right)}\right)\right]/\left(t-1\right)=4.527\;10^{-7}$ when *t* = 1200 and $MSL_{\left(1200\right)}-MSL_{\left(0\right)}=1\;m$. The acceleration of mean sea level by this model is 2*c*_3_ = 0.13 mm/yr^2^, which compares to satellite altimetry showing a climate-driven acceleration of global mean sea level of 0.084 ± 0.025 mm/yr^2^ over the last 25 yr [29] and 0.4–0.8 mm y^-1^ century^-1^ in the British Isles [30]. The exponential model gave an acceleration of 0.015 mm/yr^2^.

This quadratic model was substituted for the exponential function in Eq 6 to forecast hourly water levels using the same Monte-Carlo procedure as was described above with perturbations to monthly and hourly water levels. The water levels were binned as described above and frequency distributions computed for the 5-yr period 2064–2068 (S6 Table in S1 File) and mapped onto the DEM.

### Data sources

Water level data recorded at NOAA’s 8665530 Charleston, Cooper River Entrance gage [31] (32° 46.8' N, 79° 55.4' W) were used in this study. All water level data, including monthly means and hourly, were relative to the NAVD 88 datum. Hourly water levels in the analysis spanned 0500 hr on 10/1/1921 to 2100 hr on 12/31/2017. Hourly data were used to calculate by month and year, the maximum water level and number of times that hourly water levels exceeded a threshold for flooding, 0.38 m above mean higher high water (MHHW) or greater than 1.17 m NAVD 88. A subset of hourly data were used to compute tidal harmonic constants for forecasting the number of hours of flooding. Monthly mean water levels starting in January 1900 were used to compute the long-term trend and to forecast future MSL. Observed monthly mean water levels were transformed by adding 1.0 to the entire series to avoid negative numbers prior to fitting an exponential model

A bare earth, 1-m digital elevation model (DEM) of the Charleston peninsula derived from lidar data flown in February 2007 was obtained directly from NOAA Digital Coast [32]. The lidar data were collected using two Photo Science ALS-50 lidar sensors at a pulse rate of 75,000 points/second, projected in NAD83 UTM Zone 17N and NAVD88 geoid 12b. The DEM represents only the bare earth surface, such that areas occupied by buildings and bridges were removed from the DEM. The minimum elevation in the DEM was -0.83 m and the maximum elevation was 12.12 m (NAVD 88). The DEM cells were classified into bins representing the proportion of time each cell was flooded during the various time periods of interest as described below (S1 Fig).

## Results

### Mean monthly sea-level from the exponential model

**Fig 1:**
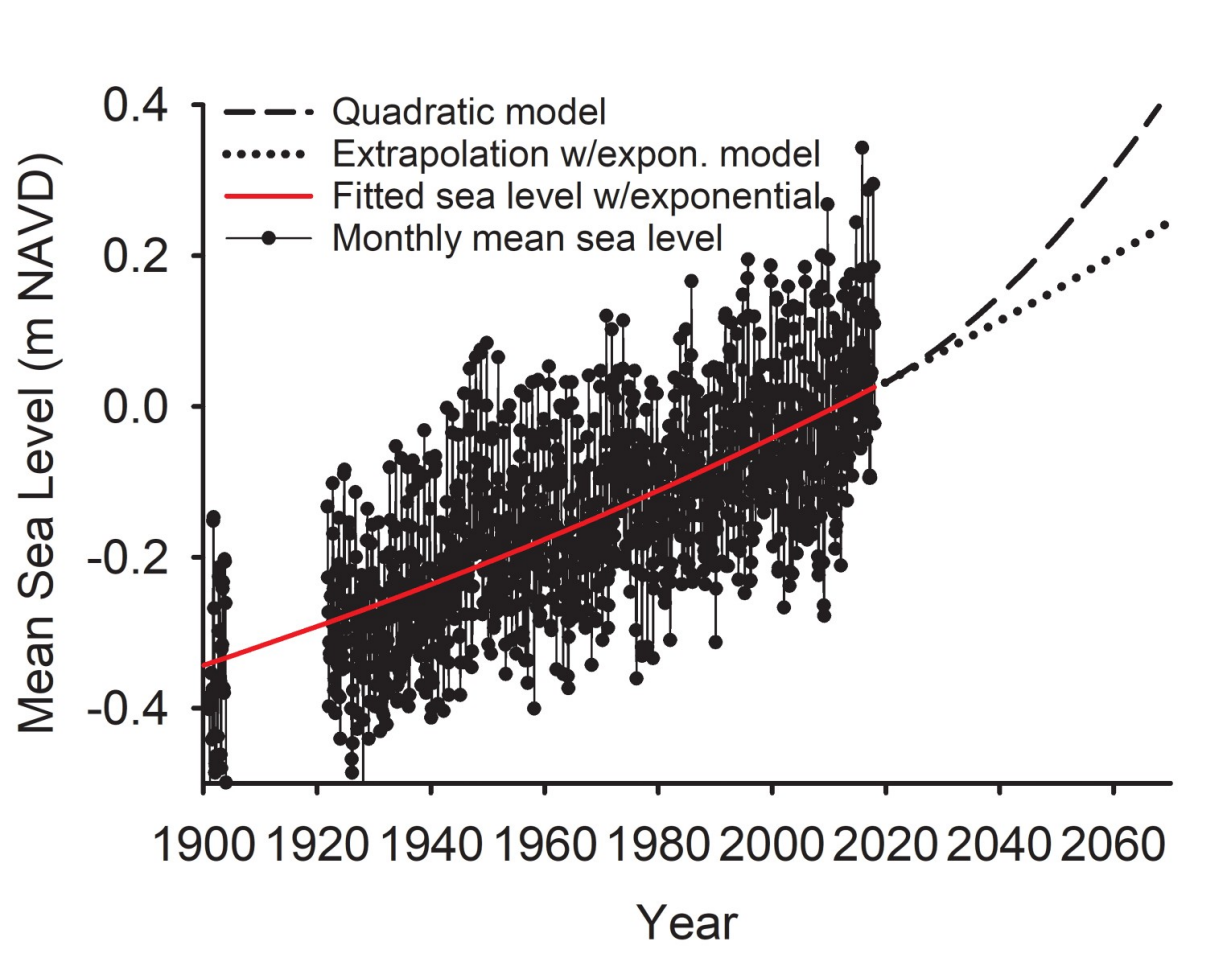
Monthly mean sea level. Observed mean monthly sea level in Charleston Harbor and the fitted exponential (Eq 1) to year 2018 (minus the seasonal and decadal harmonics: MSL_(t)_ = (MSL_(0)_ +1) e^(*r* t)^ -1, RMSE = 0.1 m, r^2^ = 0.47). Also shown is the forecasted mean sea level to the year 2068, and a forecast of a SLR scenario (quadratic model) that assumes a 1 m rise in 100 years.

The annual rate of SLR, from the first derivative of the exponential, with coefficients in Eq 1 equates to 2.4 mm/yr in 1900 and 3.4 mm/yr in year 2000, slightly ahead of NOAA’s linear trend analysis of 1901 to 2020 data that results in a 3.32 mm/yr rate [33]. If the exponential trend continues, MSL will rise another 0.22 m in the next 50 yr, rising at a rate of 0.5 cm/yr in 2069 (Fig 1), and by 0.5 m and 0.6 cm/yr by 2118, assuming that the present exponential trend continues. The quadratic model with a century of acceleration to 1 m would rise by 0.41 m at a rate of 1 cm/yr by 2069.

**Fig 2:**
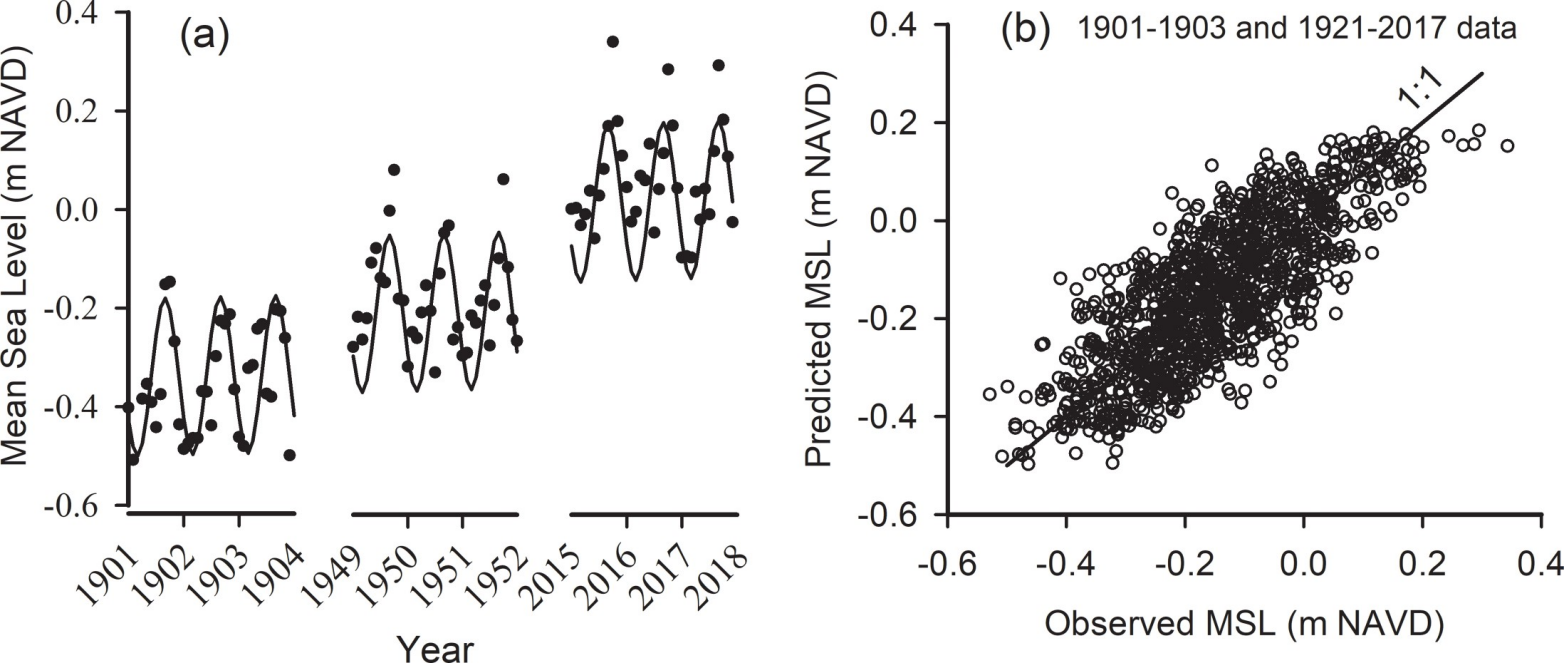
Observed and predicted monthly water levels. (a) Detail of observed (•) and fitted (**―**) monthly mean sea level, from Eq 1, for three time periods spanning the full time series. Eq 1 parameters MSL_(0)_, *r*, and *p*_*1*_ (S1 Table in S1 File) were all significant at p<0.0001. Parameter *a*_*1*_ was entered as a constant 0.16 m (see text). (b) Scatter plot of predicted versus observed monthly MSL (r^2^ = 0.62, RMSE = 0.09, n = 1233, p<0.0001).

The addition of the solar annual cycle to the exponential trend accounts for much of the seasonal variation in mean water level. As shown in detail (Fig 2), a fit of Eq 1 with this cycle to the entire time series captures the long-term trend and seasonality quite well. The r^2^ from the fit of Eq 1 improved to 0.62.

Least-squares fits of Eqs 1–4 to the monthly mean MLW, MSL, MHW, and maximum water level also returned significant results. Parameter values with significance levels are shown in S1 Table in S1 File. All were significant to p<0.0001. The estimate of the average height of monthly maximum water level above monthly MHW (w_x_) was 0.46 ± 0.003 m (approx. Std Err). A fit of model Eq 4 returned an r^2^ of 0.57 with an RMSE of 0.1 m.

The mean amplitude (from the annual maximum minus minimum monthly MSL) of the solar annual cycle was 0.159 ± 0.034 m (1900–2017, n = 100). The cycle is somewhat asymmetrical. Average maximum monthly water level occurred in September (month 9.6 ± 1), while lowest monthly water level was in February, but with considerable variation (month 1.8 ± 2). Consequently, the greatest number of nuisance flood events should occur around September.

Comparisons of unperturbed model output (Eqs 1–4) of monthly means from 2018 and 2019 with observed monthly means (data outside the training set) are in good agreement (S2 Fig). Phase plane plots of these comparisons show the seasonal changes that occurred, and in general the observed and predicted monthly means cycle around a 1:1 line. Averaged over the 19 months from January 2018 through July 2019, predicted and observed means (±1 SD) for maximum, mean high, mean, and mean low water were 1.31 ± 0.14 m, 0.81 ± 0.07 m, 0.05 ± 0.08 m, and -0.81 ± 0.09 for observed water levels, respectively, and 1.23 ± 0.11 m, 0.77 ± 0.11 m, 0.0 ± 0.11 m, and -0.84 ± 0.11 m for predictions, respectively. The RMSEs were 0.16 m for maximum water level, and 0.11 for the others.

### Mean monthly sea-level from the Monte-Carlo simulation

**Fig 3:**
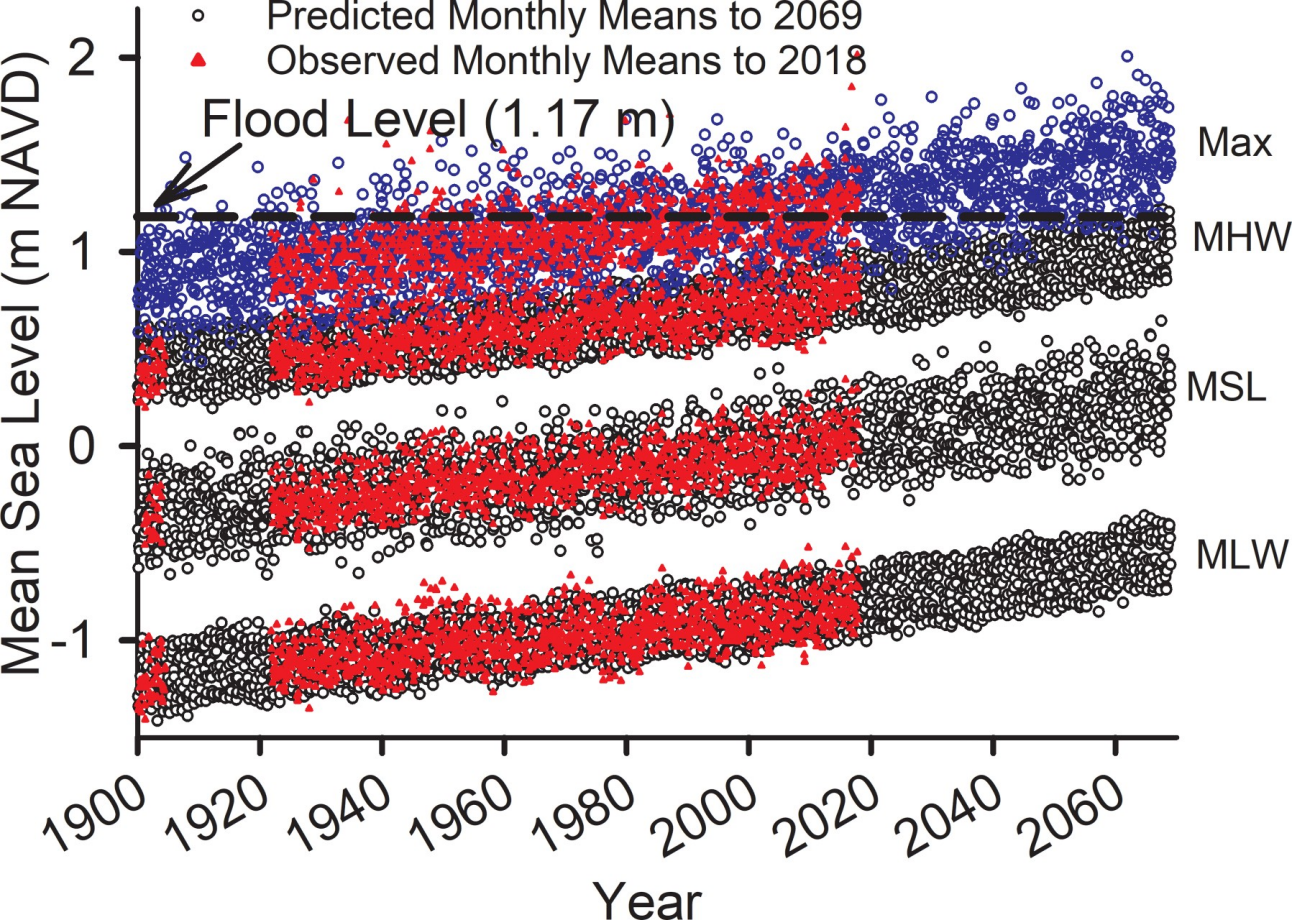
Observed and predicted monthly water levels. Observed and predicted monthly water levels with MLW, MSL, MHW and monthly maximum water level (from Eqs 1–4) with seasonal and decadal harmonics forecasted to year 2068 using a Monte-Carlo procedure with additive monthly and daily perturbations from the residuals of the model fits.

The Monte-Carlo forecast of monthly maximum water level shows that by 2060 nuisance flooding will occur during all months of the year (Fig 3). However, even now, maximum monthly water levels exceed the nuisance flood level during about 6 months of the year. By 2060 there will be some months when MHW exceeds the nuisance flood level, and during those times the flood level will be about 0.5 m above the nuisance level, because w_x_ = 0.46 (Eq 4) above MHW. Flooding will occur about 6.5% of the time during the 2046–2050 period and 8.2% of the time during the 2064–2068 period (S6 Table in S1 File).

### Annual days of nuisance flooding

**Fig 4:**
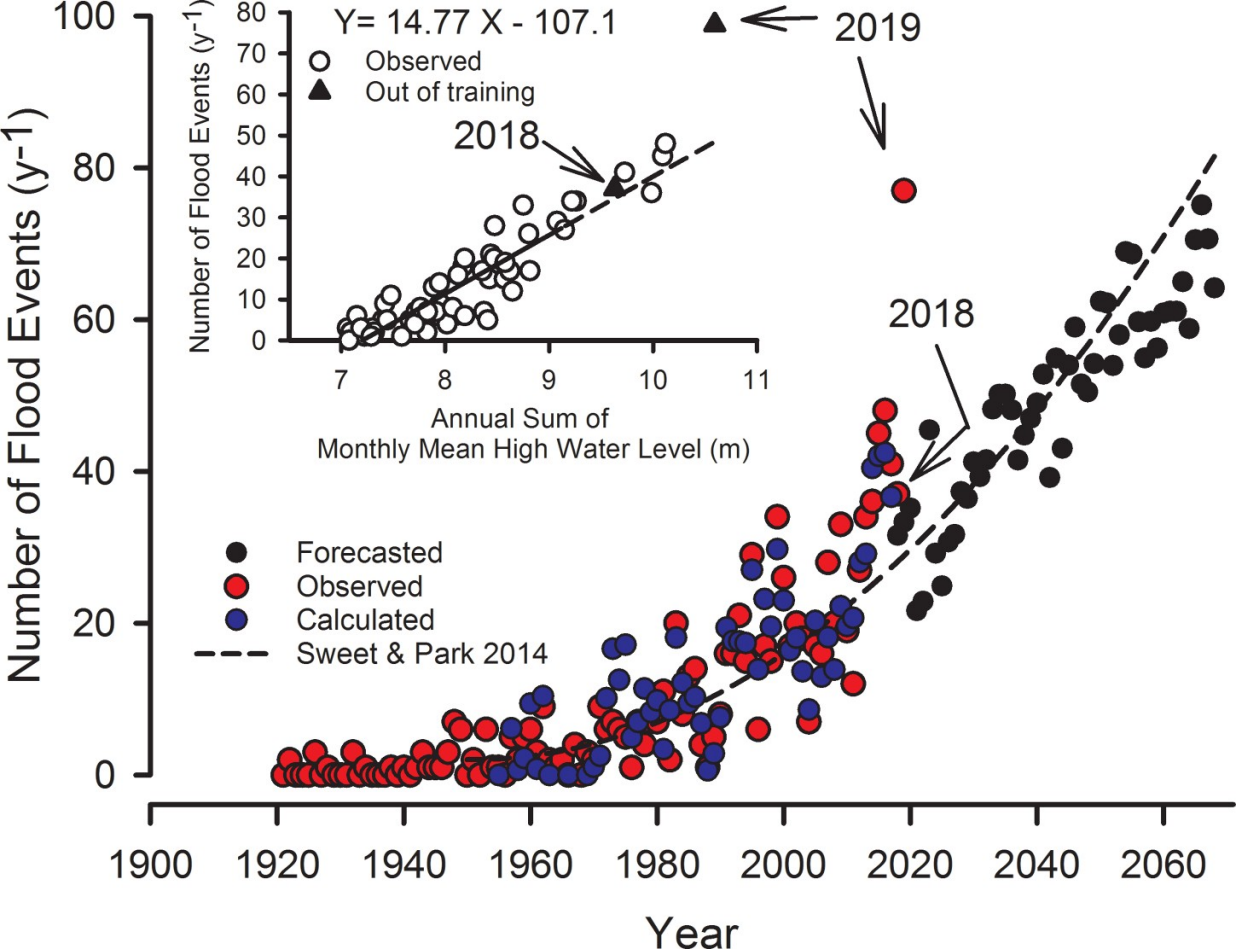
Annual observed and predicted flood events. The annual number of observed and forecasted flood events (*NF*) as a function time. The forecast was derived from the empirical relationship between *NF* and the annual sum of monthly MHW, shown in the inset, where $NF=14.77\sum_{1}^{12}MHW-107.1$ (R^2^ = 0.83, p<0.0001, N = 58). Calculated NF are from observed monthly MHW data, and the forecasted flood events are from forecasted monthly MHW (Eq 2). Also shown (-—-) is NF from the quadratic model of Sweet and Park [7].

The logic of the annual flooding-days model is that nuisance flooding likely will occur when the monthly MHW exceeds a threshold, and then the number of flooding days in any month should be proportional to monthly MHW. Extrapolating this to an annual time frame, the annual sum of monthly MHW also is likely to be proportional to the frequency of daily flooding on an annual basis. In fact, nuisance flooding has occurred when the annual sum of monthly MHW exceeded 7 m, with a slope of 14.8 floods per year per meter of $\sum_{1}^{12}MHW_{t}$ (Fig 4 inset). This simple model accounted for 84% of the variance in flood events through 2018 (Fig 4). The number of annual flood events was forecasted to year 2068 from the prediction of MHW (Eq 2 and Fig 3) and a Monte Carlo simulation with monthly perturbations. In the final 5-yr period the mean number of annual floods was 67.8 ± 6.4 (± 1 SD), i.e. there will be flooding during roughly 19% of the days in a year.

Two years of additional observed flooding data (2018 and 2019) outside the training set are also shown, and the number of flooding days during 2019 (77) was considerably higher than the prediction (50) (Fig 4 inset). NOAA’s estimate of flood frequency during 2019 was even higher, at 89, probably because they use 6 min data and we used hourly data. We do not know why the flood frequency in 2019 was so much greater than the predicted level, but it illustrates the point that the model is only an abstraction of the conditions that result in flooding such as wind speed and direction, and variation in ocean circulation [34, 35] that can modify the proportionality constant, *c*_*1*_.

Our flooding-days model (M-R model) compares favorably with the Sweet-Park quadratic model for Charleston (Fig 4), though the former has a better fit to observed data (r^2^ = .87 vs 0.63) owing to the fact that the Sweet and Park model (S-P model) is simply a function of time. Where the two models diverge greatly are in their forecasted durations. The quadratic S-P model projects 144 hr/yr of flooding by 2068, or 1.65% of time, the M-R model projects 718 hrs/yr or 8.2% of time (S6 Table in S1 File). The hours per day of flooding on days of flood in the S-P model is nearly constant at 1.77 while flooding hours per day in the M-R model rises from 6.7 during 2014–2017 to about 10 hours during 2065–2068, i.e. average flood duration on days of flood is expected to rise from 6.7 to 10.2. It should rise because the tidal harmonic curve is rising above a fixed plane. Using a polynomial function Moftakhari et al. [36] projected up to 315 h per year of flooding in Charleston by 2050 under RCP 8.5. The M-R model predicts 521 h of flooding by 2050 from just the current exponential rise. Paraphrasing Rahmstorf and Vermeer [37], modelling flood duration and frequency as a simple function of time is not physical, because time is not a direct cause of flooding. Ultimately, however, all of these models are directly or indirectly functions of time.

### Hourly water level

**Fig 5:**
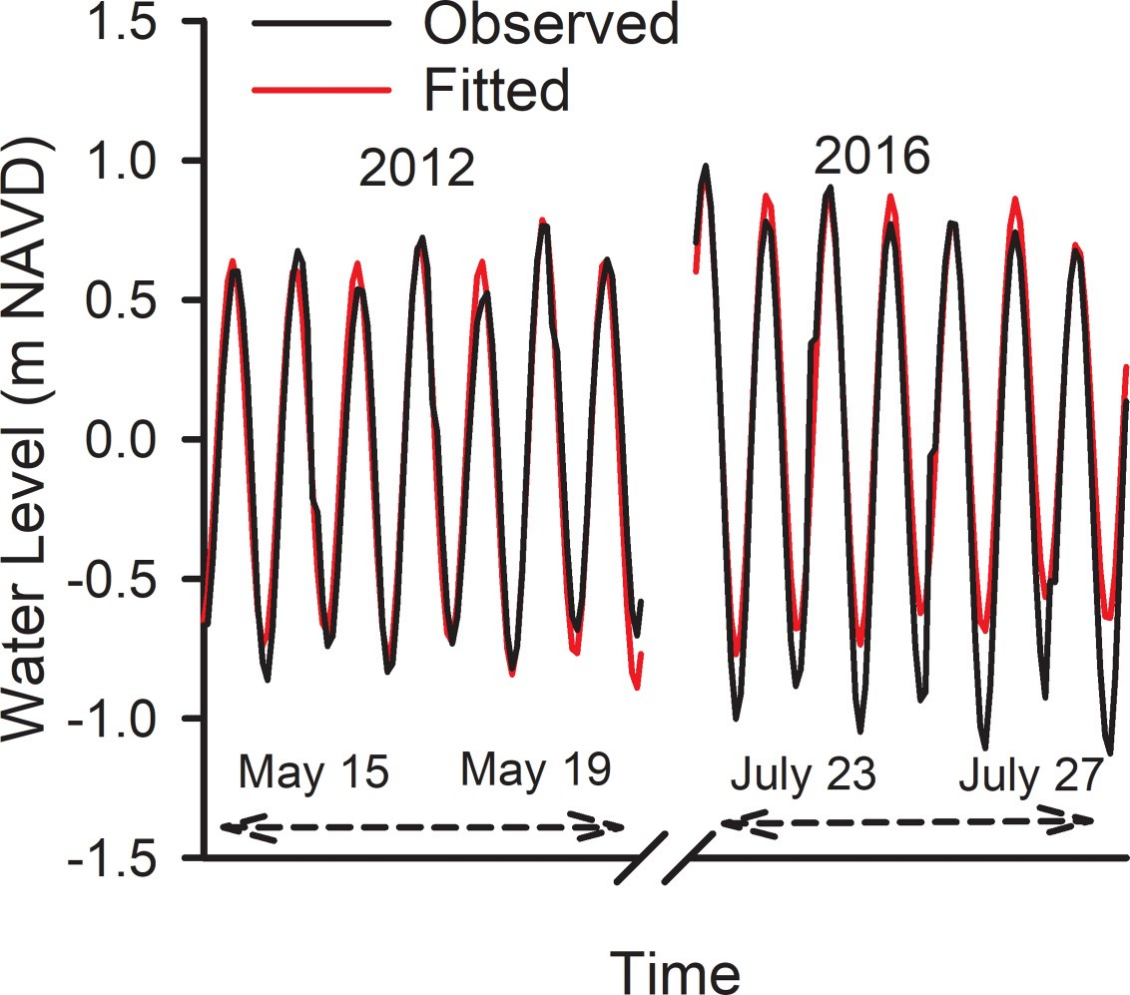
Hourly time series of water level. Details from the hourly time series of water levels. Predictions are from Eq 6. Observed data from NOAA station 8665530.

Harmonic regressions for the stepwise fits to all 10 years of hourly water levels accounted for 87% to 89% of the variability for each annual period, and all parameter estimates were statistically significant (p<0.0001, S5 Table in S1 File). A plot of arbitrary portions of the hourly time series from the period May 15 to May 19, 2012 and July 23 to July 27, 2016 showed good agreement between observed and predicted water levels (Fig 5). The RMSE of the entire 5-yr time series had an RMSE of 0.28 m. A linear regression of the predicted hourly water levels, MSL_(h)_ in Eq 6, against observed water levels from years 2012 and 2016 had a slope of 0.87 (r^2^ = 0.88), and the entire hourly series from 2011 to 2018 had a slope of 0.88 (r^2^ = 0.88). This compares to NOAA’s predicted versus verified water levels from 2012 and 2016 that had a slope of 0.95 (r^2^ = 0.93) (S3a Fig).

### Flood duration and spatial extent

Our forecasts of flood duration and spatial extent are based alternatively on the exponential extrapolation of Charleston sea level or a quadratic extrapolation of the NOAA intermediate SLR scenario with added noisy tidal harmonics. This is equivalent to the analysis of nuisance flooding in Boston employed by Ray and Foster [38], except that their forecasts were based on a linear extrapolation of sea level as well as an intermediate-high scenario with a deterministic model of the tidal harmonics superimposed.

Integrations of Monte-Carlo forecasts of the hourly water levels (Eq 6) to year 2068 gave inundation times expressed here as fractions of time water levels exceed different elevations. For example, for the 5-yr period 2012–2017, water level exceeded the 1.17 m nuisance threshold 0.8% of total time (S4 Table in S1 File). The exponential forecast predicted a 2012–2017 exceedance time of 3.1%. The exceedance time is predicted to rise to 6.5% by 2046–2050, and then to 8.2% by 2064–2068 (S4 Table in S1 File). The quadratic model (Eq 7) predicted an exceedance time of 14.2% by 2064–2068. Exceedance times for water levels of 1.55 m or higher were predicted by the exponential model to rise from 0.3% (2012–2017) to 1% (2046–2050) and 1.3% (2064–2068). At the 1.55 m or greater level, the quadratic model forecasted an increase to 3.1%.

**Fig 6:**
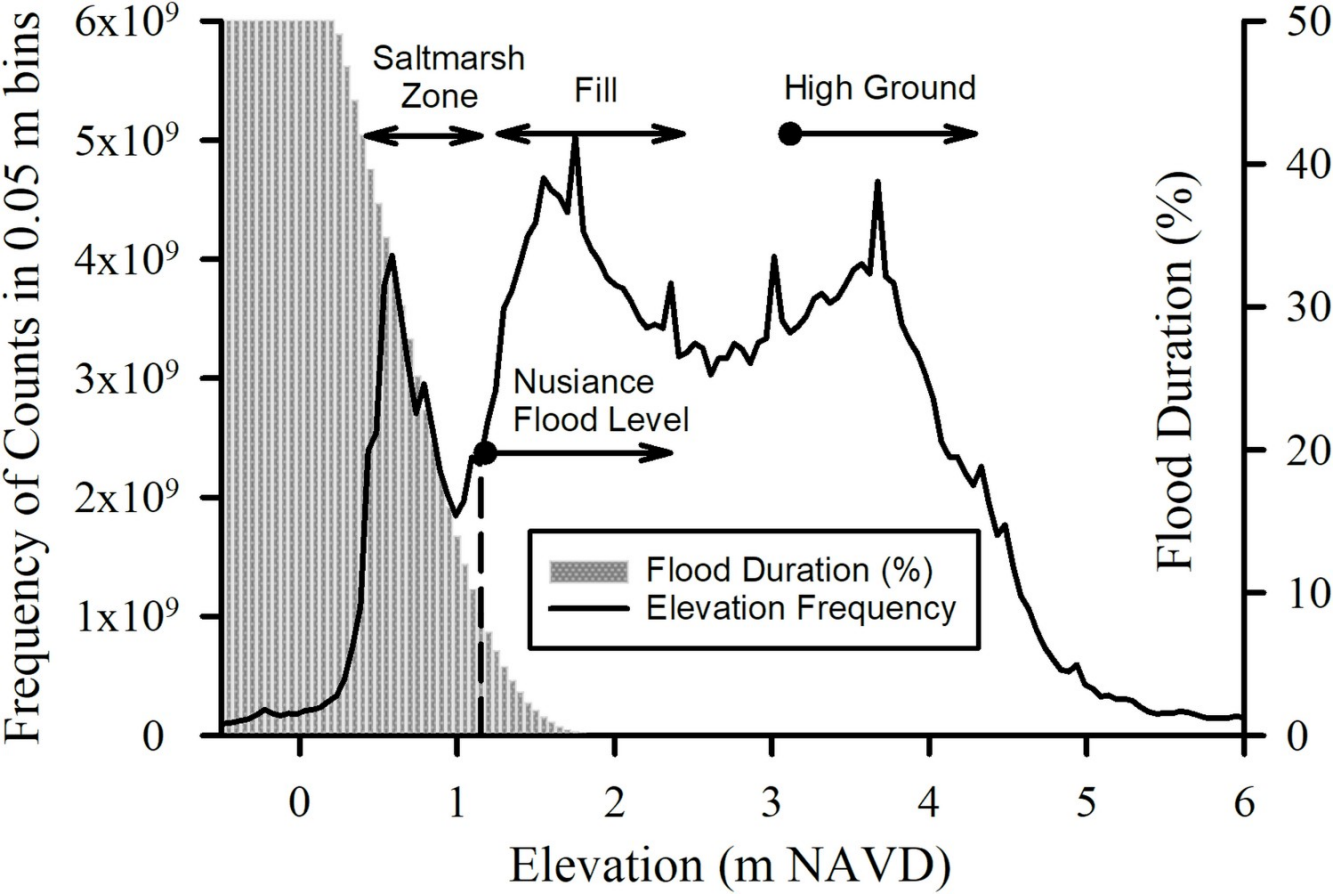
Frequency distribution of surface elevation. Frequency distribution of Charleston peninsula elevations in 0.05 m bin classes and the exponential model forecast of flood duration (% of time flooded) during years 2064–2068 as a function of elevation.

**Fig 7:**
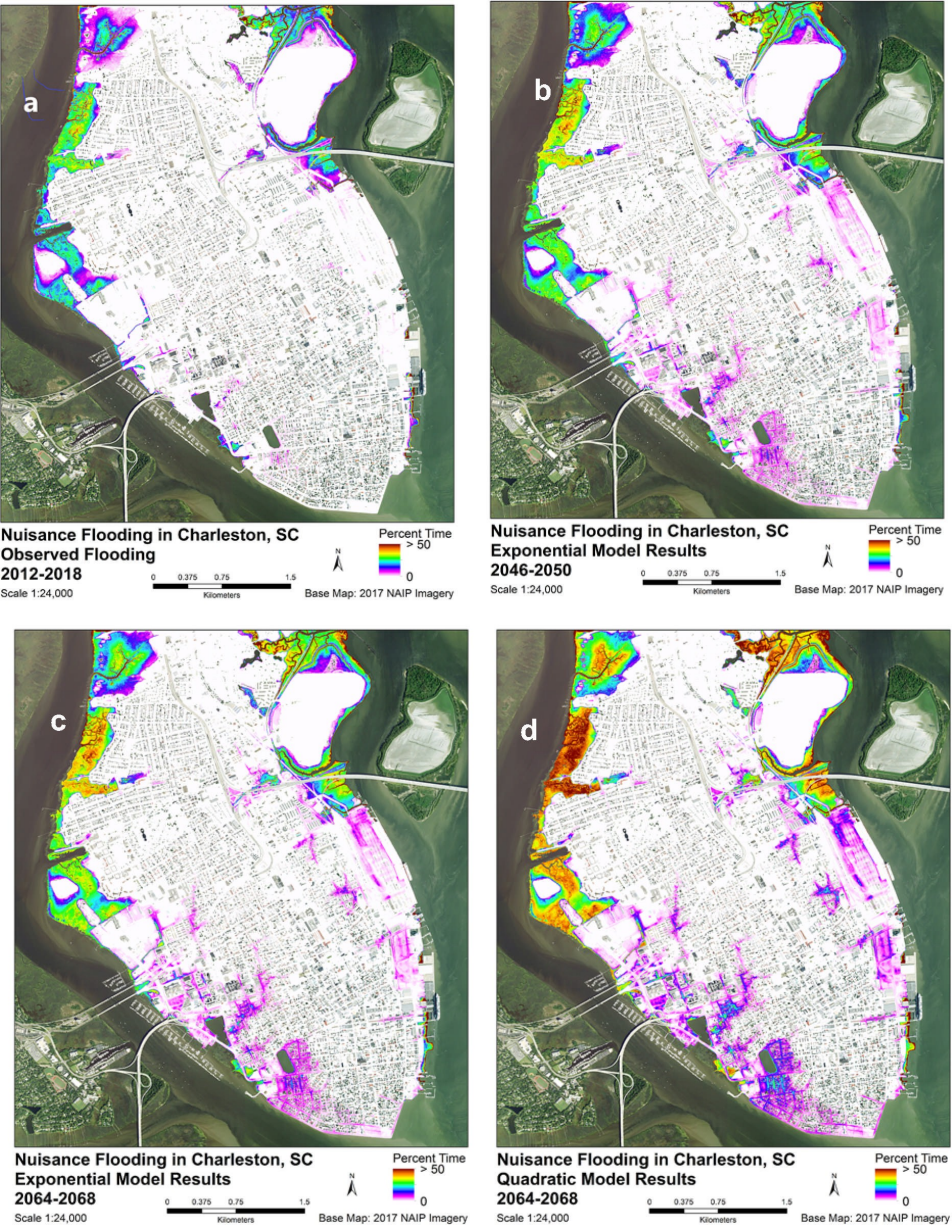
Spatial distribution of flood duration. Current spatial distribution of flood duration on Charleston Peninsula (a), and forecasted flood duration (proportion of time flooded) in 2046–2050 (b) from the exponential model of sea-level rise, and forecasted flood duration in 2064 to 2068 from the exponential model (c) and quadratic model (d). Basemap: 2017 NAIP Imagery acquired from USDA-FSA-APFO.

The frequency distribution of Charleston peninsula surface elevations shows three distinct peaks or modes (Fig 6). A peak frequency at elevation 0.5 m, having the lowest frequency of the three, corresponds to salt marsh. There is a 0.9 m minimum corresponding to a transition to the next higher level, followed by a rapid rise in frequency to the next peak elevation at 1.5 m. This elevation is a common land surface, within the nuisance flood zone, and it probably represents land reclaimed by filling of marshes and creeks. The third peak at 3.7 m is above the current nuisance zone and appears to be safely higher that the forecasted, 2068 maximum monthly water level by either the exponential or quadratic model. Nuisance flooding appears to be limited currently and until 2060 to former marshland and creeks (Fig 7) that were filled in earlier centuries to make room for development.

## Discussion

This paper adds to a growing body of evidence for acceleration of sea-level rise. The processes that affect relative sea-level rise like subsidence, tectonic movements, and rising temperature can have additive or opposing effects and may explain why acceleration of rising sea level has not been detected in SE U.S. Atlantic stations. Detection also depends on the length of the record, the data starting date, noise, and the ability to filter decadal-scale cycles [19, 37] and choice of model. Using a quadratic model, for example, nether [7] nor [39] were able to detect acceleration in the tide records from Charleston Harbor.

We show from the long record of tide data recorded in Charleston that sea level is rising at an exponential rate and accelerating at about 0.018 mm/yr^2^. This is small compared to recent satellite altimetry data showing that global mean sea level has accelerated to 0.084 ± 0.025 mm/yr^2^ [29] and, from tide records, compared to 0.083 to 0.15 mm/yr^2^ along the Atlantic East Coast [39]. If the trends continue, MSL will rise 0.65 m by 2100 [29] or from 0.48 to 0.71 m by 2050 [39]. Like previous analyses [7, 39], the fit of a quadratic model to Charleston, SC tide data was not significant (S3 Table in S1 File). However, the exponential model in the present paper was significant and projects a 0.24 m rise by 2068. Extrapolation of a quadratic model resulted in a rise of 0.39 m by 2068, assuming a 1 m rise in a century. These are significantly lower than rates derived from the Northeast Atlantic by Boon [39], but greater than his 2050 projection for Charleston. However, even a 0.24 m rise will subject 4.23 km^2^ or 20% of the Charleston peninsula to monthly flooding (Fig 3).

Charleston is going to be difficult to defend from rising sea level because it is surrounded on three sites by water (Fig 7). Areas most susceptible to nuisance flooding in Charleston include the residential area to the east of Colonial Lake, the western portion of the residential area south of Broad Street, developed areas around a hospital and university, residential areas along Gadsden Creek on the western side of the peninsula, the industrial area just south of the Cooper River Bridge extending west to the Charleston Museum on Meeting Street, the tourist area surrounding the Aquarium and Gadsdenboro Park, and Waterfront Park on the eastern side of the Peninsula. The increase in exposure to nuisance flooding will affect all sectors of the economy–from industrial to education, and from tourism and recreation to residential and transportation.

Flooding is a high priority item with Charleston City Council and the Office of the Mayor, with $240 million in drainage projects allocated to date. But the cost to implement solutions for Charleston is estimated to be at least $2 billion and is likely to go higher [40]. Charleston’s annual visitor population of 6.9 million represents an opportunity for raising the needed revenue. The per capita cost on the basis of that population is modest and would not be excessive if appropriated over a period of years by a policy similar to a hospitality tax. However, we would call it a climate tax, and it should be applied to residents and visitors alike. Further, we would advocate a climate tax that would be invested and accrue in a fund–a climate growth fund, in the same way a pension fund is invested. The climate fund could be used for a number of climate-related problems in future years, like combating tropical and infectious disease, in addition to coastal defenses. Humans have taxed the Earth’s climate, and the time has come for a climate tax in order to insure human welfare.

## Supporting information

S1 Fig Base map of Charleston peninsula. (TIF)

S2 Fig Phase plane plots of observed and predicted monthly mean water levels. (TIF)

S3 Fig Comparisons of model and NOAA predicted and verified hourly water levels. (TIF)

S1 File (RTF)
