# Supplementary figures and images for: Past, present, and future nuisance flooding on the Charleston peninsula
Source: PLoS One. 2020 Sep 18;15(9):e0238770. doi: 10.1371/journal.pone.0238770 (PMC7500608; doi:10.1371/journal.pone.0238770)

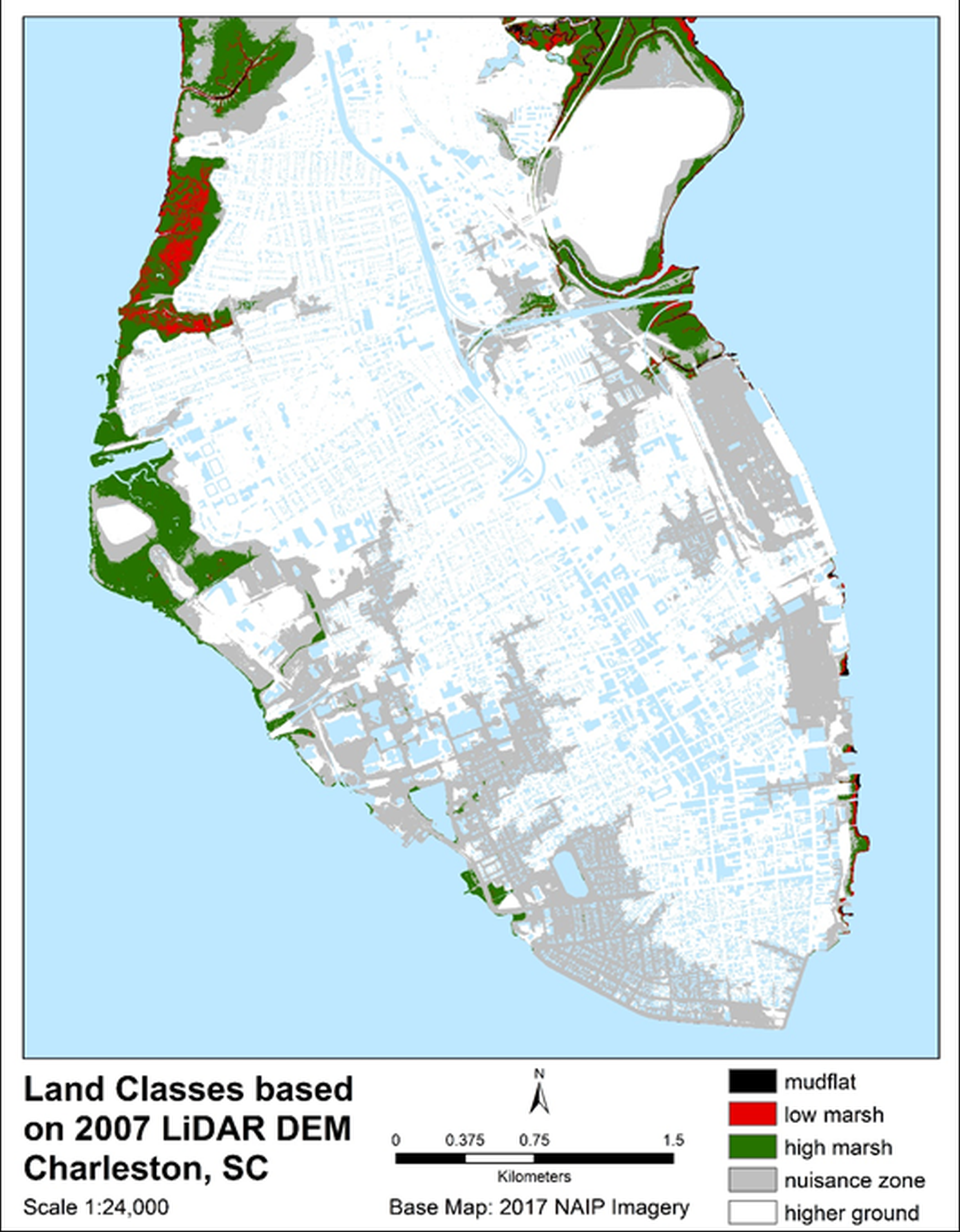

Supplement: S1 Fig — (TIF) [file pone.0238770.s001.tif]

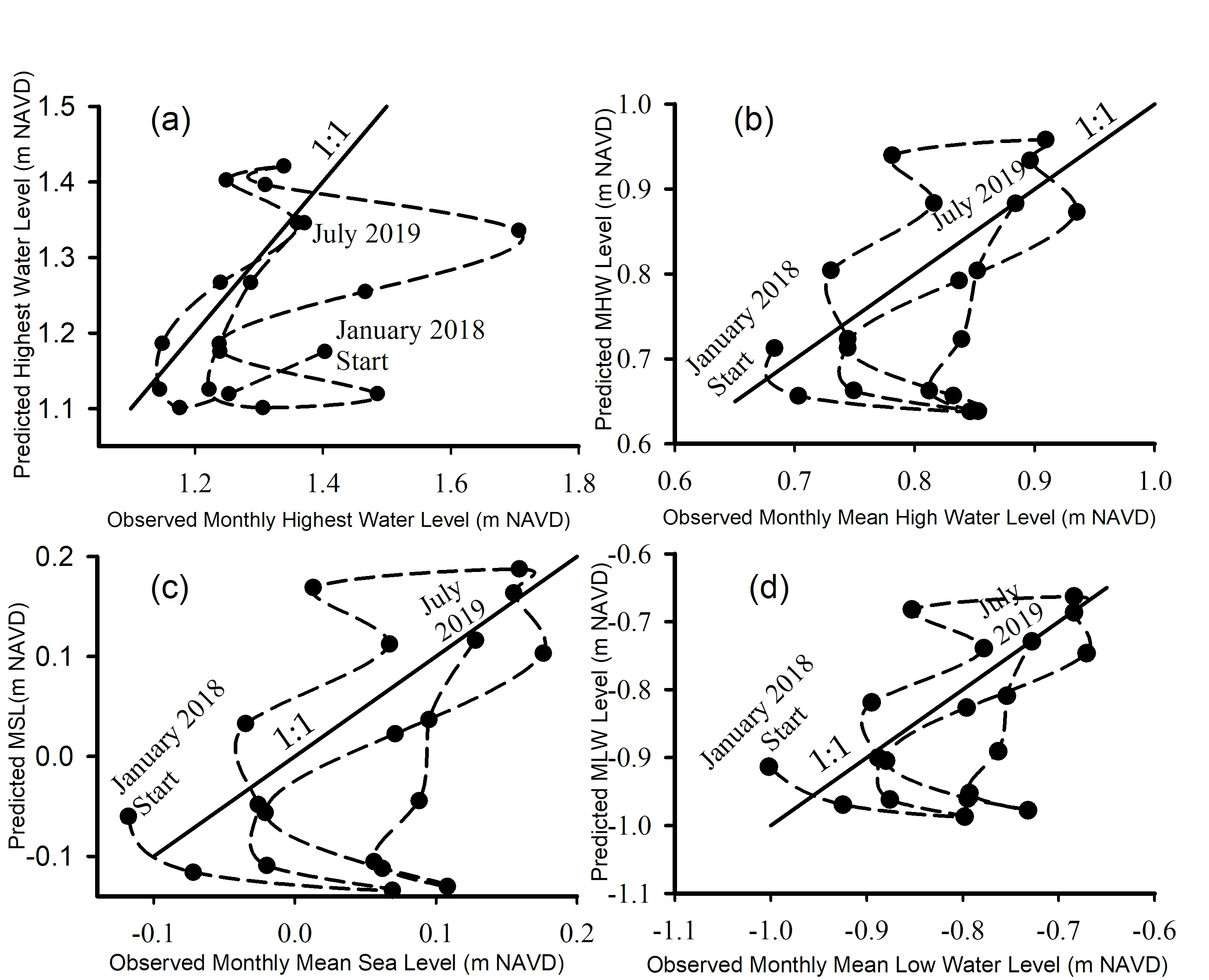

Supplement: S2 Fig — (TIF) [file pone.0238770.s002.TIF]

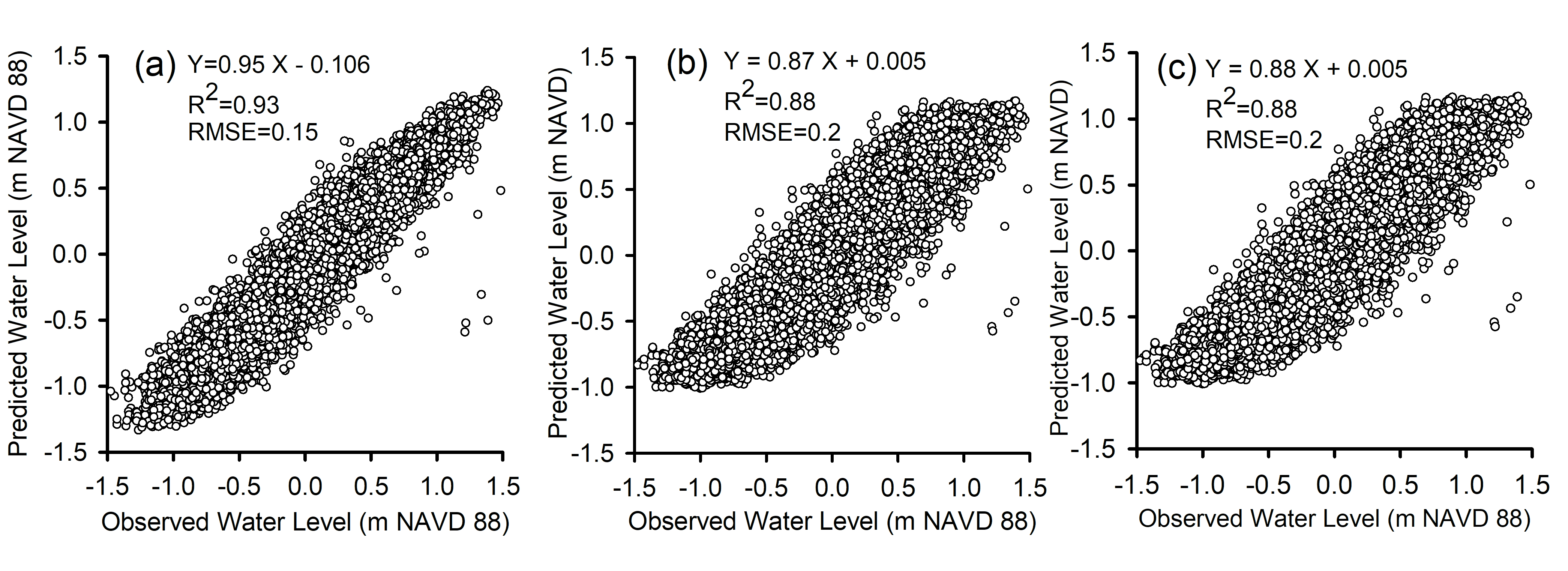

Supplement: S3 Fig — (TIF) [file pone.0238770.s003.TIF]
