# Supplementary material for: Past, present, and future nuisance flooding on the Charleston peninsula
Source: PLoS One. 2020 Sep 18;15(9):e0238770. doi: 10.1371/journal.pone.0238770 (PMC7500608; doi:10.1371/journal.pone.0238770)
Supplement: S1 File — (RTF) [file pone.0238770.s004.rtf]

S1 Fig. Base map of Charleston peninsula with classified elevations based on a 2007 DEM (NOAA digital coast). Mudflat was defined as ground surface below -0.07 m NAVD88, low marsh as lying between -0.07 and 0.45 m NAVD88, and high marsh between 0.45 and 0.83 m NAVD88. The nuisance zone is any elevation exposed to flooding based on the model predictions and is between 0.83 and 1.85 m NAVD88. Higher ground was defined as any surface above 1.85 m NAVD 88.  Areas with a turquoise color are no data, or areas outside the DEM (water, buildings, elevated roads).  Basemap: 2017 NAIP Imagery acquired from USDA-FSA-APFO: https://www.fsa.usda.gov/programs-and-services/aerial-photography/imagery-programs/naip-imagery/.  

S1 Table.  Nonlinear OLS parameter estimates of constants used to model monthly MSL, MHW, MLW and monthly maximum water level from the exponential model (Equations 1-4).                                                         	
Parameter	Estimate	Approx Std Err	t Value	Approx	
				Pr > |t|	
r	0.0003	0.000	39	<.0001	
MSL(0)	-0.353	0.005	118	<.0001	
p1	3.1793	0.025	127	<.0001	
p2	0.0235	0.067	94	<.0001	
aL	0.6720	0.001	1231	<.0001	
aH	0.5973	0.001	1155	<.0001	
CL	18.635	0.001	375	<.0001	
a2	0.0197	0.001	28	<.0001	
a3	0.0264	0.001	33	<.0001	
wx	0.6227	0.005	116	<.0001	


S2 Fig. Phase plane plots of observed and predicted monthly mean water levels from January 2018 through July 2019. Predicted water levels were not fitted and are unperturbed and calculated from Eqs. 1-4.


S2 Table.  Summary statistics of the residuals from the exponential, monthly mean model of MSL, MHW, MLW and maximum monthly water levels (m).	
Residuals	N	Year of Earliest Data	Mean	Std Dev	Minimum	Maximum	
MSL	1190	1901	0.000	0.095	-0.288	0.272	
MHW	1209	1900	-0.001	0.095	-0.260	0.307	
MLW	1209	1900	0.001	0.106	-0.287	0.343	
Maximum 	1151	1921	0.000	0.143	-0.514	1.409	


S3 Table. Nonlinear OLS parameter estimates of quadratic fit to monthly sea level (1921-2017 inclusive) : MSL = w0 + c1t + c2t2

Nonlinear OLS Summary of Residual Errors 

Equation
DF Model
DF Error
SSE
MSE
Root MSE
R-Square
Adj R-Sq
Label

MSL
3
1211
12.5323
0.0103
0.1017
0.4938
0.4930
MSL

	
Nonlinear OLS Parameter Estimates 

Parameter
Estimate
Approx Std Err
t Value
Approx
Pr > |t|

w0
-0.36037
0.0133
-27.02
<.0001

c1
0.000244
0.000036
6.78
<.0001

c2
2.056E-8
2.184E-8
0.94
0.3467

	


S4 Table.  Results of flood models tested.  Shown are the parameter values (a, b, or c), and the annual number of predicted nuisance flood events (NOF) at different time points, including the mean sea level predicted in 2068 from the exponential and quadratic models.  Model 6 was used for the nuisance flood forecasts shown in the paper.  The rationale for the excluded data filter is apparent in the Fig. 4 inset.	
			Model Statistics	Model Parameters	Annual NOF predictions	
Model Tested		† Additional Filter for Data Excluded 	n	R2	RMSE	a	b	c	From 2017 Obs Sea Level 	From 2068 Exp SLR Mod	From 2068 Quad SLR Mod	
1.		NOF =a (monthly MSL) + b		84	0.78	5.4	10.39	24.86		30	55	173	
2.		NOF=a exp(b(monthly MSL))+b		84	0.87	4.2	24.7	0.816		36	263	1509	
3.		NOF = c (monthly MSL)+b	(mthly MSL) < -2	57	0.84	4.7	14.96	27.9		35	71	103	
4.		NOF = c (monthly MHW)+b		84	0.77	5.6	10.1	-68		30	51	76	
5.		NOF= a exp(b(monthly MHW))		84	0.83	4.7	0.021	0.768		38	181	1234	
6.		NOF = a(monthly MHW)+b	(mthly MHW) < 7	62	0.84	4.8	14.3	-103		36	66	101	
7.		NOF = a MSL		84	0.78	5.4	124.8	24.9		30	55	77	
8.		NOF= a exp(b MSL)		84	0.87	4.2	24.7	9.79		36	261	1508	
9.		NOF= a + b (1+MSL)c		84	0.9	3.7	-5.49	15.554	7.919	37	149	413	
10.		NOF = a MSL+b 	Ann. MSL< -0.1	37	0.87	4.2	233.2	29.1		38	85	127	
11.		NOF = a MHW+b		84	0.77	5.6	121.7	-68		31	51	77	
12.		NOF= a exp(b MHW)		84	0.84	4.7	0.02	9.22		35	168	1180	
13.		NOF= a + b (1+MHW)c		84	0.87	4.2	-6.35	16.77	7.24	38	115	351	
14.		NOF = a MHW+b	MHW < 0.625	47	0.84	4.8	202.7	-125.7		39	73	116	
† All annual mean sea level data through 2017 with an annual maximum MHW < 0.65 m were excluded 		
				Independent variables used in NOF computations			
			year	MHW	MSL	MHW	MSL	MHW	MSL	NOFobs‡		
grand means (1950-2017 inclusive)	From NOAA data:	2017	9.727	0.465	0.8105	0.039	0.145	0.134	41		
		From NOAA data	2018	9.629	0.439	0.8024	0.037	0.132	0.132	37		
		From NOAA data	2019	10.595	1.527	0.8829	0.127	0.217	0.223	77		
MSL (m NAVD 88)	-0.095	From Expon. SLR Mod:	2068	11.8	2.9	0.98	0.241	0.314	0.336			
MHW	0.666	From Quad. SLR Mod:	2068 	14.298	5.04	1.1915	0.42	0.526	0.515			

S1 Methods. Tidal Harmonics
Hourly tide levels were from harmonic regressions fitted to hourly data spanning 2008 through 2017.  We fitted a model accounting for five important tidal constituents:
Eq. S1 	MSL(h) stepwise = M2(h) + S2(h) + K1(h) + O1(h) + Sa(h) + Wmean  
where h is the cumulative hour from January 1 to the end of the year, and Wmean is the mean annual water level (m) for the year, and Sa is the solar annual component as defined in Eq. 1.   The fitted tidal constituents were:
The principal lunar constituent: 		M2= a4 sin(2h/12.4206 + p4)
The principal solar constituent: 		S2= a5 sin(2h /12 + p5)
The diurnal constituent: 			K1= a6 sin (2h/23.9345 + p6) 
The lunar diurnal constituent: 		O1= a7 sin(2h /25.8193 + p7)
The solar annual constituent:		Sa= a8 sin(2h /(365.25×24) + p8)
The hourly model (Eq. S1) was fitted in a stepwise manner to hourly water levels for each of 10 annual time series from 2008 to 2017, and the fitted constants for amplitudes and phase shifts averaged (Table S5).  The harmonic regressions for all 10 years accounted for 87% to 89% of the variability in hourly data for each year and all parameter estimates were statistically significant (p<0.0001).  Scatter plots comparing NOAA hourly predictions from Charleston 8665530 from the years 2012 and 2016 and Eq. S1 predictions from those same years and for years 2011 through 2017 are shown in S3 Fig. 
Next, the annual hourly time series from 2012 through 2017 were concatenated and the model fitted again with known amplitudes and phase shifts, from the means of the stepwise procedure, and with the addition of the exponential function (MSL(0) e(r t)) having coefficients derived by fitting Eq. 1 (main text).  This was fitted to a continuous time series of hourly data from 2012 to 2017:

   Eq. S2 	MSL(h) = .6527e (.000321 t) + M2 + S2 + K1 + O1+ Sa  + Wcorr

Where t and h are the cumulative months (starting 1345 months) and hours (starting 2177448 hours) since January 1900, respectively.  Parameter Wcorr was the intercept (0.027 m NAVD  0.001 SE) for the January 2012 water level. The constants in the exponential were from the analysis of monthly mean sea levels (Eq. 1 main text).  
To forecast hours of flooding we used Eq. S2 to simulate the tidal cycle by Monte-Carlo simulation with a probability distribution based on the standard deviation of the hourly (å(h) mean 0, SD 0.297 m) and monthly residuals (å(t) mean 0, SD 0.095 m).  Each month a new monthly permutation was computed and each day a new hourly permutation was computed and added to the deterministic model output.
Hourly water levels from the Monte Carlo forecasts of years 2012-2018, 2046-2050, and 2064-2068 were binned into 0.05 m groupings, and cumulative frequency distributions were computed (SAS 9.4 Proc FREQ)  from the binned water levels (Table S6).  Each bin contained the percentage of total hours that water levels reached that elevation or higher during each sequence of years.  From the frequency distributions we calculated the proportion of time that water level exceeded the threshold flood level of 1.17 m NAVD. based on a report by Sweet et al. (2014) who identified the nuisance flood level in Charleston as a water level exceeding 0.38 m above MHHW.  


S5 Table. Nonlinear OLS parameter estimates of harmonic constants used to model hourly water level from the 2012 to 2017 data, inclusive.	
Parameter	Estimate	Approx Std Err	Period (h)	t Value	Approx	
	Phase Shift (rad)				Pr > |t|	
M2	2.1895	0.00229	12.4206	889.52	<.0001	
S2	0.9996	0.0151	12	66.29	<.0001	
K1	3.0374	0.0179	23.9345	169.48	<.0001	
O1	0.702263	0.0251	25.8193	28.02	<.0001	
Sa	2.775826	0.0112	8766	247.40	<.0001	
	amplitude (m)					
M2	0.7770	0.0005		1254.25	<.0001	
S2	0.1187	0.0005		1168.75	<.0001	
K1	0.1011	0.0467		402.76	<.0001	
O1	0.0729	0.0007		30.77	<.0001	
Sa	0.0252	0.0007		33.73	<.0001	


S3 Fig. (a) NOAA predicted and verified hourly water levels from years 2012 and 2016. (b) Eq. S2 computed and verified water levels from nonlinear regressions to hourly data from years 2012 and 2016 and (c) from years 2011 to 2017, inclusive.  Willmott's refined dimensionless index of agreement [31] for the NOAA data set in (a) is 0.86 compared to 0.84 in (b).  A value of 1.0 is a perfect match.

S6 Table. Observed and forecasted flood durations as a percentage of total time that water levels attain or exceed the elevations in column 1.  	
	Frequencies (% of total hours)	
Elevation Bin NAVD (m)	Observed	Exponential Model	Quadratic	
	2012-2017	2012-2017	2046-2050	2064-2068	2064-2068	
0.5	27.8	26.1	33.6	37.0	45.2	
0.55	24.5	23.7	31.3	34.5	42.9	
0.6	21.4	21.4	28.8	32.0	40.5	
0.65	18.3	19.2	26.3	29.5	38.2	
0.7	15.2	17.0	23.8	27.0	35.6	
0.75	12.4	14.9	21.4	24.7	33.2	
0.8	9.7	12.8	19.2	22.4	30.6	
0.85	7.5	10.9	17.0	20.0	28.1	
0.9	5.7	9.2	14.8	17.5	25.8	
0.95	4.2	7.6	12.9	15.4	23.4	
1	3.0	6.3	11.1	13.4	21.0	
1.05	2.1	5.1	9.3	11.5	18.8	
1.1	1.4	4.0	7.8	9.7	16.4	
1.15	0.8	3.1	6.5	8.2	14.2	
1.2	0.5	2.4	5.4	6.8	12.3	
1.25	0.3	1.8	4.4	5.6	10.6	
1.3	0.2	1.3	3.5	4.5	8.9	
1.35	0.1	1.0	2.8	3.6	7.5	
1.4	0.1	0.7	2.2	2.8	6.1	
1.45	0.0	0.5	1.7	2.2	4.9	
1.5	0.0	0.4	1.3	1.7	3.9	
1.55	0.0	0.3	1.0	1.3	3.1	
1.6		0.2	0.8	1.0	2.4	
1.65	0.0	0.1	0.6	0.7	1.9	
1.7		0.1	0.4	0.5	1.5	
1.75	0.0	0.1	0.3	0.4	1.1	
1.8		0.0	0.2	0.2	0.8	
1.85	0.0	0.0	0.1	0.2	0.6	
1.9	0.0	0.0	0.1	0.1	0.4	
1.95		0.0	0.1	0.1	0.3	
2	0.0	0.0	0.0	0.0	0.2	
2.05		0.0	0.0	0.0	0.1	
2.1			0.0	0.0	0.1	


Supplemental references


Willmott C, Robesonb S, Matsuuraa K,  A reﬁned index of model performance Int. J. Climatol. 2012; 32: 2088–2094. DOI: 10.1002/joc.2419
